# Low-cost calcium fluorometry for long-term nanoparticle studies in living cells

**DOI:** 10.1038/s41598-020-69412-1

**Published:** 2020-07-28

**Authors:** Connor L. Beck, Clark J. Hickman, Anja Kunze

**Affiliations:** 1 Department of Electrical and Computer Engineering, Montana State University, Bozeman, Montana, 59717 USA; 2 Present Address: Department of Physics, North Carolina State University, Raleigh, NC 27695 USA

**Keywords:** Nanoparticles, Imaging and sensing, Endocytosis

## Abstract

Calcium fluorometry is critical to determine cell homeostasis or to reveal communication patterns in neuronal networks. Recently, characterizing calcium signalling in neurons related to interactions with nanomaterials has become of interest due to its therapeutic potential. However, imaging of neuronal cell activity under stable physiological conditions can be either very expensive or limited in its long-term capability. Here, we present a low-cost, portable imaging system for long-term, fast-scale calcium fluorometry in neurons. Using the imaging system, we revealed temperature-dependent changes in long-term calcium signalling in kidney cells and primary cortical neurons. Furthermore, we introduce fast-scale monitoring of synchronous calcium activity in neuronal cultures in response to nanomaterials. Through graph network analysis, we found that calcium dynamics in neurons are temperature-dependent when exposed to chitosan-coated nanoparticles. These results give new insights into nanomaterial-interaction in living cultures and tissues based on calcium fluorometry and graph network analysis.

## Introduction

Imaging calcium dynamics in and between neurons is essential to analyse neural signalling and to better understand how drugs, metabolites, and neural treatments impact the plasticity of signalling in neural networks. Commercially available techniques to record calcium activity rely on the induction of a calcium-dependent fluorescent sensor and fluorescent-based high-resolution microscopy for both in vivo and in vitro applications^1^. High-resolution optical microscopy, e.g., two-photon or confocal microscopy, has revealed valuable knowledge about subcellular calcium signalling^2^. These imaging modalities, however, usually require expensive optical setups, integrated laser systems, and high computing power for fast image processing, which is often limited in remote, low-resource capacity regions. Furthermore, adverse photo tissue interactions^3^ still bind optical microscopy techniques to in vitro operations using cultured brain slices or neural networks grown from dissociated brain tissues. These tissues and neuronal networks grown from cultures require an incubator environment that mimics the physiological concentration of oxygen, carbon dioxide, humidity, and temperature. Some of the weaknesses of traditional optical microscope systems may limit point-of-care systems in low-cost healthcare environments. These weaknesses, however, can be overcome through small-size digital microscopy integrated into customized incubation systems.

Over the last decade, digital microscopy has seen growth in the form of lens-free imaging platforms and small-scale single-lens digital imaging platforms^4^. Both imaging platforms offer low-cost, high-speed fluorescent imaging capabilities for a large field of view that permits imaging of time-resolved cell dynamics or point-of-care disease screenings^5,6^. Furthermore, both imaging platforms are small enough to fit into portable incubator systems allowing for long-term imaging of cell dynamics over several hours, days, or weeks. Using a three-dimensional printed lens-free video microscopy platform, Kesavan et al*.* were able to monitor cell growth kinetics, cell motility continuously, and cell death of mesenchymal stem cells, bone, and skin cancer cells for up to 90 h at high-content (> 100,000 measurements per experimental condition)^7^. Reconstruction of cell features, however, required computationally intensive holographic image processing methods and access to high-resource setting cell incubation methods. A combination of a long-term, low-cost, live-cell imaging and incubation system has been introduced by Walzik et al*.*^8^. The authors used webcam-based digital microscopy in an in-house designed incubator to capture cell proliferation of kidney cancer cells (HEK293) for up to 48 h^8^. Rajan et al*.* built a portable upright digital imaging platform with additional capabilities for extracellular electrophysiological recording^9^. Both systems, the webcam-based and upright digital microscopy, allow for bright and darkfield illumination, but not for fluorescent-probe-based sensing.

Here, we demonstrate the capability of using off-the-shelf fluorescent digital microscopy^10^ in combination with coloured light-emitting-diode illumination and white-light illumination to capture calcium dynamics in several hundreds of neurons simultaneously in a low-cost and portable incubation system. We validated the robustness and portability of our system through two experimental sets. The first set demonstrates the capability of long-term image acquisition through monitoring temperature-dependent calcium dynamics in HEK293 cells in a lab-extern cell culture facility and primary cortical neurons grown in our lab. The second set validates fast-scale, short-term image acquisition through monitoring temperature-dependent calcium influx, and efflux events under reduced carbon-dioxide conditions. Precisely, we were able to characterize slow, long-term calcium dynamics in primary cortical neuron cultures based on (a) temperature-dependent temporal changes in calcium signalling, (b) calcium events associated with cell death, and (c) fast-scale spatiotemporal changes in synchronous calcium dynamics associated with the uptake of chitosan-coated nanoparticles. This low-cost, portable, and easy to assemble long-term imaging platform can expand fluorescent imaging of neuronal cell dynamics to low-resource environments, field settings, and even classrooms. Hence it has the potential to expand knowledge-gaining and next-generation neuro-tool development to a broader academic spectrum.

## Results and discussion

### Portable live-cell imaging system for low-cost fluorometry

**Figure 1:**
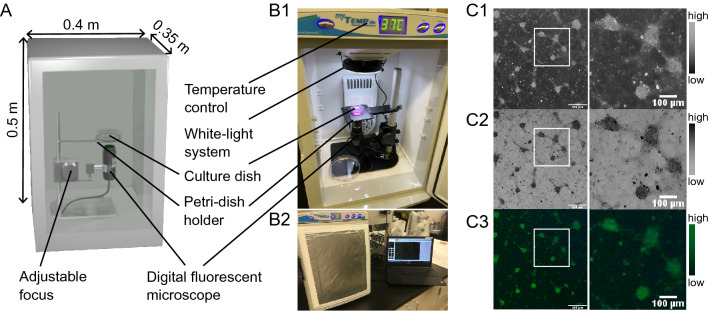
The portable integrated live-cell fluorescent imaging system to study calcium dynamics in mammalian cells. (**A**) Conceptual design of a low-cost, a light-weighted imaging system for continuous monitoring of live-cell activity using fluorescent probes. (**B1**) Incubator set up with an adjustable biological sample holder, digital fluorescent microscopy, and white-light system. (**B2**) Bench-top setup of the imaging system for multi-lab usage. (**C1**) Acquired bright field grey-scaled image shows neuronal cell clusters grown from dissociated primary neurons during week two. (**C2**) Corresponding inverted grey-scaled image. (**C3**) Green-fluorescent image of Fluo4 AM loaded neurons. Scale bar = 300 µm.

Our live-cell fluorescent imaging system consists of four parts: a portable, compact bench-top incubator, a digital microscope with connection to a portable computational station, a white-light LED ring, and an adjustable petri dish holder (Fig. 1A). All four parts are off-the-shelf components and were chosen for a fast and easy assembly that required only a few modifications to the incubator system. Hence the resultant, low-cost imaging system allows for high reproducibility in a training/classroom setting or a low-resource environment. The bench-top assembly of the incubator is shown in Fig. 1B1 and B2. The incubator has integrated temperature control and the possibility to be upgraded to regulate carbon dioxide (CO_2_) levels. The digital microscope provides software-controlled switchable blue and yellow light-emitting diodes (LEDs) for 480 nm and 575 nm excitation with an integrated emission filter between 510 and 610 nm. A white-light LED ring was installed at the top of the incubator to add bright field imaging. A representative white-light cell culture image taken with primary cortical neurons is shown in Fig. 1C1, with its inverted version shown in Fig. 1C2. Figure 1C3 shows the corresponding green-fluorescent signal with 480 nm excitation of the Fluo-4 AM loaded neurons. Image contrast of these images can be assessed through histogram plots, which are shown in supplementary data (Fig. S6, Fig. S7, see supplementary files).

The complete live-cell imaging system weighs 8.1 kg**,** has dimensions of 35 cm × 40 cm × 50 cm, and costs below US$ 2000. Our imaging solution is an improvement of existing long-term imaging systems^5,8–10^, as it combines portability with temperature control and digital calcium fluorometry for low-cost live-cell studies without the need for 3D printing, expertise in computer-automated-design, and skills in assembling. The small footprint and lightweight characteristics of our imaging system make live-cell fluorometry and portability between research and classroom space possible and accessible.

### Digital fluorescent imaging characteristics

**Figure 2:**
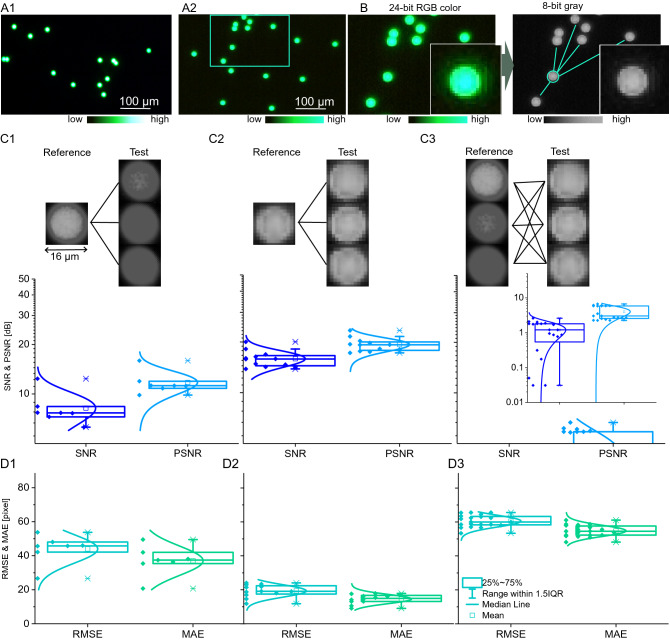
Digital fluorescent imaging is comparable to high-end fluorescent microscopy at the single-cell level. (**A1**) Magnified fluorescent images of green-fluorescent beads (15.65 µm) imaged with a high-end fluorescent optical microscope (40x) and (**A2**) the digital microscope (220x). (**B**) Large-scale comparison of fluorescent image uniformity and signal-to-noise ratio was performed on grey-scale, 8-bit images, which were down-sampled from multi-colour 24-bit RGB colour images. (**C1**–**D3**) Comparison tests were performed on individual green-fluorescent beads in relation to near-distance (< 1 mm) neighbouring beads based on a signal-to-noise ratio (SNR), a peak signal-to-noise ratio (PSNR), a mean absolute error (MAE), and a root-mean-square error (RMSE). (**C1**–**C2**) Boxplots show distribution of image quality based on SNR and PSNR for (**C1**) the high-end fluorescent optical microscope (n = 10), and for (**C2**) the low-cost digital microscope (n = 10). (**C3**) Randomized inter-comparison of SNR and of PSNR of fluorescent bead appearance between high-end optical and low-cost digital microscopy (n = 10). (**D1**–**D2**) Boxplots show distribution of image quality based on MAE and RMSE for (**D1**) the high-end fluorescent optical microscope (n = 10), and for (**D2**) the low-cost digital microscope (n = 10). (**D3**) Randomized inter-comparison of SNR and PSNR of fluorescent bead appearance between high-end optical and low-cost digital microscopy.

To assess the optical characteristics and limitations of our digital fluorescent imaging system, we used Dragon green fluorescent beads with a diameter of 15.65 µm and compared their optical appearance between the digital fluorescent microscope against a commercially available high-end, fluorescent optical microscope. Figure 2A1 shows the green fluorescent beads in a 24-bit, RGB image taken with a green-emission filter (mercury lamp excitation) under the high-end optical microscope (40×, 2.0 megapixel). In contrast, Fig. 2A2 shows the same beads imaged with the digital microscope in a 24-bit RGB image (220×, 1.3 megapixels). The fluorescent digital microscope provides two-dimensional images from a CMOS camera in a field-of-view with 1280 × 1024 pixel array (1574 µm × 1180 µm), resulting in an imaging resolution of 1.352 pixels per micrometre at highest magnification (220×). The high-end optical microscope provides 1.25 pixels per micrometre (with 10× objective) and 3 pixels per micrometre (with 20× objective). The higher optical zoom, however, reduces the field-of-view for the optical system down to 668 µm × 668 µm. Hence, the imaging resolution of the digital fluorescent microscope is comparable to the high-end optical microscope and, therefore, suitable for fluorometry at the cellular level and similar to image resolutions reported in Yang et al*.*^11^. The large field-of-view of the digital system furthermore provides an advantage for cell network studies.

Next, we characterized the spatial homogeneity of the optical appearance of the beads within the field of view. For this comparison, 24-bit images were downsampled to 8-bit grey-scale images (Fig. 2B) and single bead images extracted (43 × 43 pixels) for signal-to-noise ratio (SNR), mean-absolute-error (MAE), peak signal-to-noise (PSNR), and root-mean-standard error (RMSE) analysis. The first test compares the spatial bead appearance within the high-end optical microscope image (Fig. 2C1), showing an averaged SNR of 8 dB and an averaged PSNR of 11.5 dB. In contrast, the second test compared images taken with the digital fluorescent microscope. These images have a slightly higher averaged SNR and PSNR of 17 dB and 20 dB (Fig. 2C2), in which a randomized comparison test between the two microscopes also confirms (Fig. 2C3). However, the PSNR and RMSE show lower values for images captured with the digital than with the high-end optical microscope, indicating higher signal uniformity within the digital image acquisition (Fig. 2D1–D3). Furthermore, the high-end microscope showed a more substantial variance in both the SNR and PSNR. This characteristic can be explained through higher sensitivity and imaging artefacts from multiple optical lenses and a longer light path. While high-end optical microscopes have the advantage of picking up signal differences at the subcellular scale, digital fluorometry provides more uniformity and lower noise sensitivity.

### Validation of long-term live-cell fluorometry through analyzing temperature-dependent calcium events

**Figure 3:**
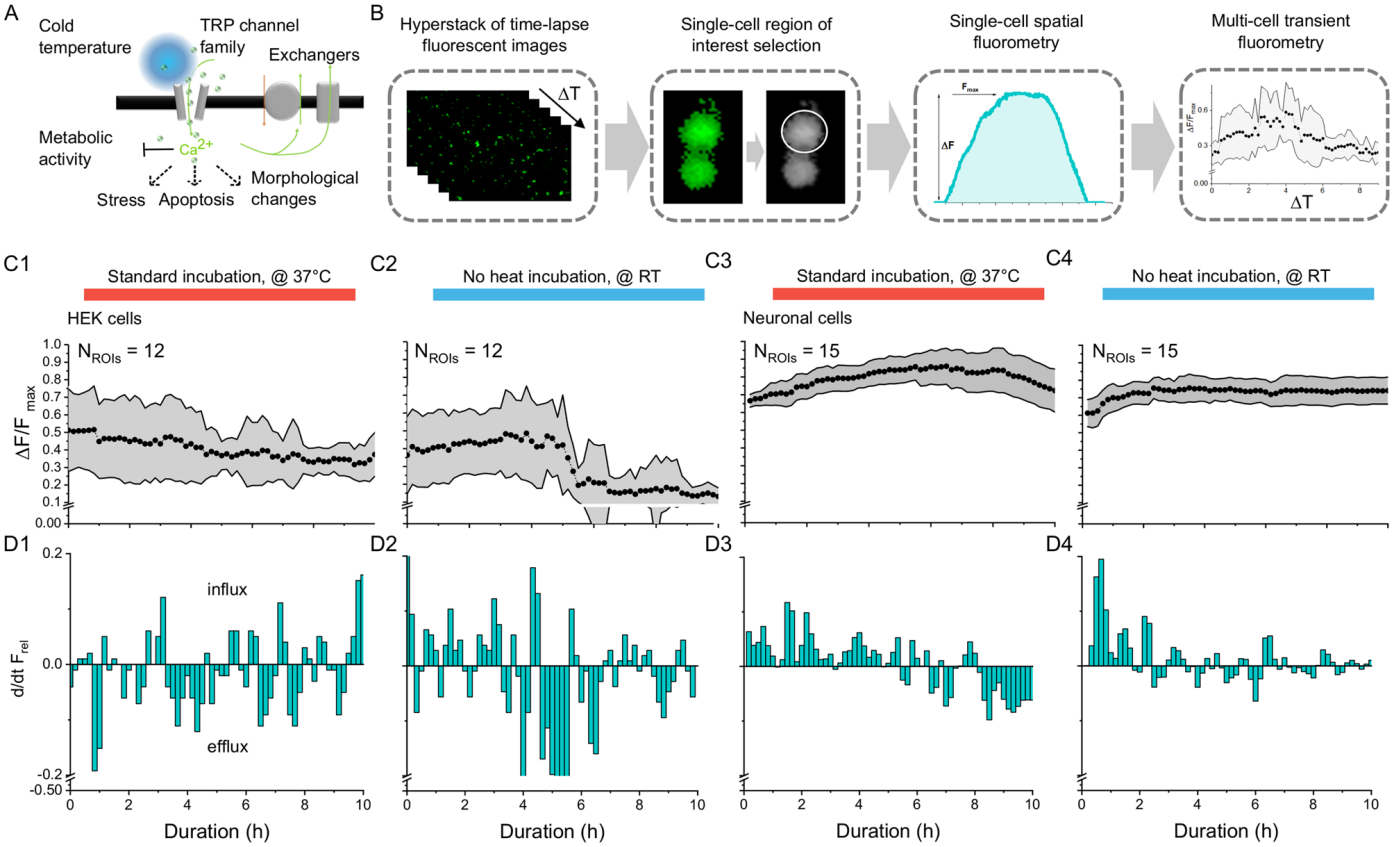
Temperature dependence on long-term fluorescent calcium imaging in mammalian cells. (**A**) HEK cells are known to express temperature-dependent calcium channels, which may trigger calcium in- or efflux through so-called TRP channel families. (**B**) Image processing workflow shows how we continuously captured fluorescent images using the digital microscope in our incubator setting for single- and multi-cell spatiotemporal calcium fluorometry and metabolic studies. (**C1-4**) Temperature-dependent changes in intracellular calcium levels in (**C1–C2**) HEK cells and (**C3–C4**) primary cortical neurons (E18, rat). (**D1–D4**) Calcium influx and efflux characteristics show higher temperature sensitivity of HEK cells versus neuronal cells.

Human embryonic kidney cells are known to express endogenous calcium channels^12^. One class of ion channels is called transient receptor potential (TRP) channels and mainly known to regulate the intake of cations under changes in temperature^13^. Within the class of TRP channels, some specific channels are more selective to regulate calcium influx (TRPV5, TRPV6) than others^14^, which is, however, beyond the focus of our study, here. TRP channels sit in the cell membrane pointing towards the extracellular space where they can sense changes in temperature, graphically sketched as a cold blue ball in Fig. 3A. Depending on the type, TRP channels can be either cold-sensitive channel (< 21 °C, TRPM8), or heat-sensitive (> 43 °C, TRPV1) to gate calcium into the cytosol^15–19^. Once activated, TRP channels allow calcium ions to enter the cytosol, which can lead to further downstream processes causing cell apoptosis, changes in metabolic activity, or cell morphology^20–25^. Hence, we used HEK cells to demonstrate the ability of our imaging system to monitor temperature-effects in long-term calcium fluorometry. To capture temperature sensation in HEK cells, we performed a 10-h, live-cell fluorescent imaging experiment using Fluo4 AM as a calcium probe in our portable incubator systems either under 37 °C with temperature control, or under room temperature (~ 20 °C, RT). Images were taken every 10 min with 1 s exposure time. From the time-lapse images, we extracted single-cell fluorescent profiles and plotted averaged changes in fluorescent activity from multiple cells over time (Fig. 3B).

HEK cells incubated with 37 °C temperature control maintained regular calcium activity, as represented by an almost constant average intensity profile over the 10 h (Fig. 3C1). Analyzing calcium efflux and influx events, as shown in Fig. 3D1, confirms physiological healthy cell behaviour. We then imaged HEK cells incubated under RT (temperature control was switched off, and the system was cooled to RT) and observed a 67% decay in fluorescence intensity between 5 and 6 h incubation (Fig. 3C2). Between 5 and 6 h, all calcium events can be attributed to calcium efflux, indicating induced cell death to prolonged exposure to temperatures below the physiological temperature level of 37 °C.

Next, we extended our cold-sensation study to primary cortical neurons, which were dissociated from embryonic brain tissues (rat, E18) and grown for 2 weeks. Based on the same calcium fluorometry used in HEK cells, cortical neurons express a higher baseline in normalize calcium signal intensity (ΔF/F_max,_ Fig. 3C1, C2 vs. Fig. 3C3, and 3C4) in their cell bodies with fewer oscillations between calcium influx and efflux events, and lower influx and efflux amplitudes (Fig. 3D1, D2 vs. Fig. 3D3, and D4). Visually comparing the period of oscillatory calcium events between neurons and HEK cells showed lower values for neurons (Fig. 3D1–D4) than for HEK cells. More stunningly, incubating neurons under room temperature for 10 h resulted in a uniform calcium profile. Calcium efflux events were present but not as significant in amplitude as in the HEK-cell experiment (Fig. 3D2, D4). This contrasting behaviour between primary cortical neurons and HEK cells may link to the different members of TRP channels. It may be likely that neurons endogenously express more heat-sensitive TRPV1 than cold-sensitive TRPM8. Variation in calcium activity due to inhomogeneity in cultured neuronal networks or the sample size is shown in Fig. S1 and Fig. S2 and can be excluded as a course of effect (see supplementary file).

### Slow, long-term changes in calcium dynamics associated with cell death

**Figure 4:**
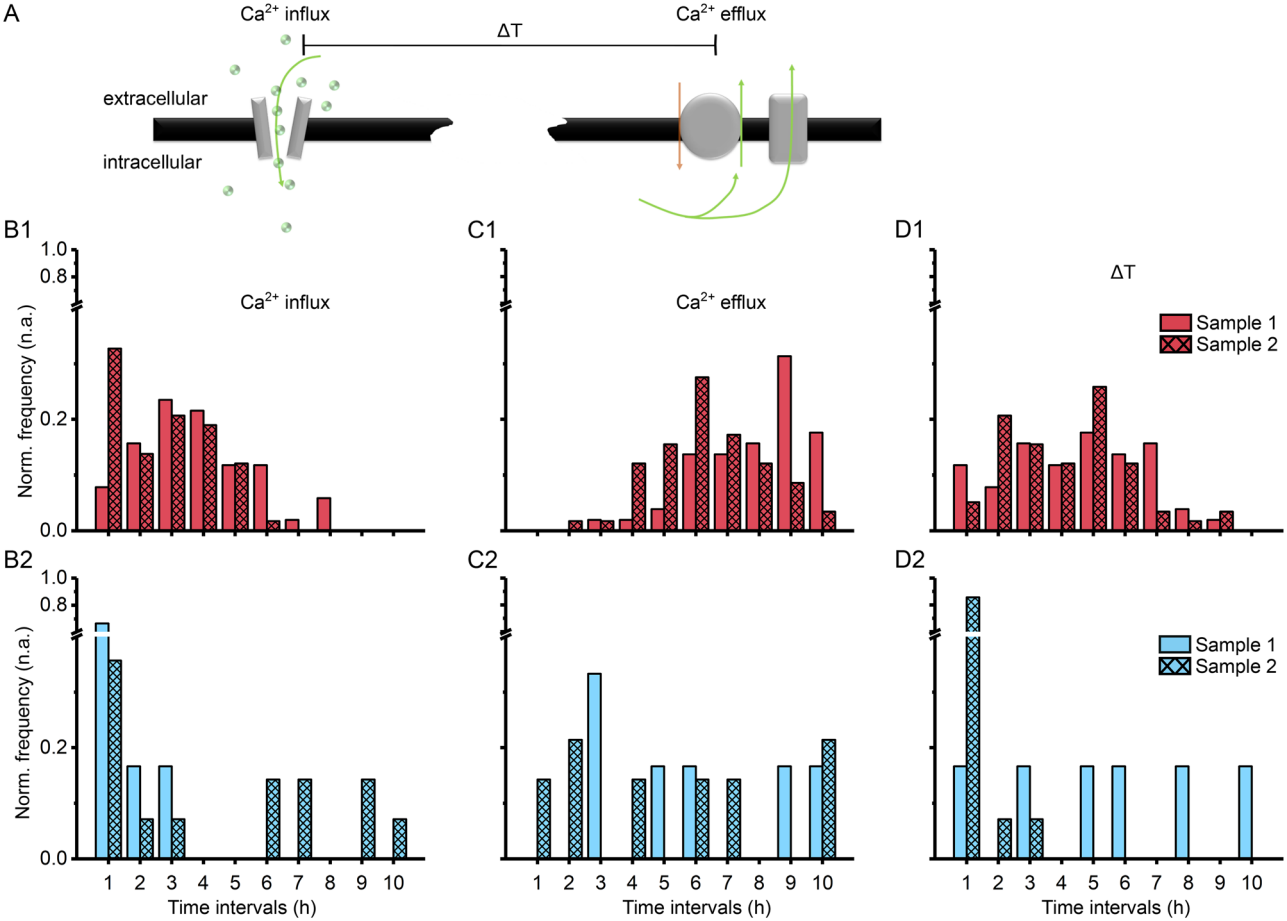
Long-term calcium fluorometry reveals temperature-dependent effects on cell death. (**A**) Differences in time-delay (ΔT) between calcium influx and calcium efflux through either channels, exchangers, or a rupture in the cell membrane may allow differentiating between cell apoptosis and cell necrosis. (**B1**–**B2**) Counts of calcium influx under (**B1**) physiological (37 °C) and (**B2**) room temperature (RT), n_cells_ = 20. (**C1**–**C2**) Counts of calcium efflux under (**C1**) physiological and (**C2**) room temperature, n_cells_ = 20. (**D1**–**D2**) Temporal differences between calcium influx and efflux under (**D1**) physiological and (**D2**) room temperature (n = 2).

One aspect of temperature-mediated processes in neurons is related to calcium signalling and its link to cell death^20–22,24,26,27^. Although our experiments suggest that neurons, when imaged at RT, show almost no decrease in average calcium fluorescent signalling, we carefully analysed our data regarding calcium signals that shown only one spike (a one-time calcium influx followed by calcium efflux) in a cell body over the whole time course. The single spike event may indicate the occurrence of cell death. For calcium spike events that occurred only once, we measured the time delay (ΔT) until the calcium efflux occurred (Fig. 4A). In Fig. 4B1 and C1, we show the distribution of neuronal cell bodies where only one spike event was detected, and when influx and efflux events occurred. Under physiological temperature and across 10 h, most of the selected cell bodies seem to die around 5 h (Fig. 4D1). In contrast to RT, 70% of the calcium influx events occurred within 1 h (Fig. 4B2), and 83% of cells also seem to die with ΔT of 1 h (Fig. 4C2, D2). These differences in ΔT between RT and 37 °C point towards a temperature-related cell death mechanism.

### Validation of fast-scale, low-cost calcium fluorometry to capture spatiotemporal differences in neuronal calcium signalling during nanoparticle interaction

**Figure 5:**
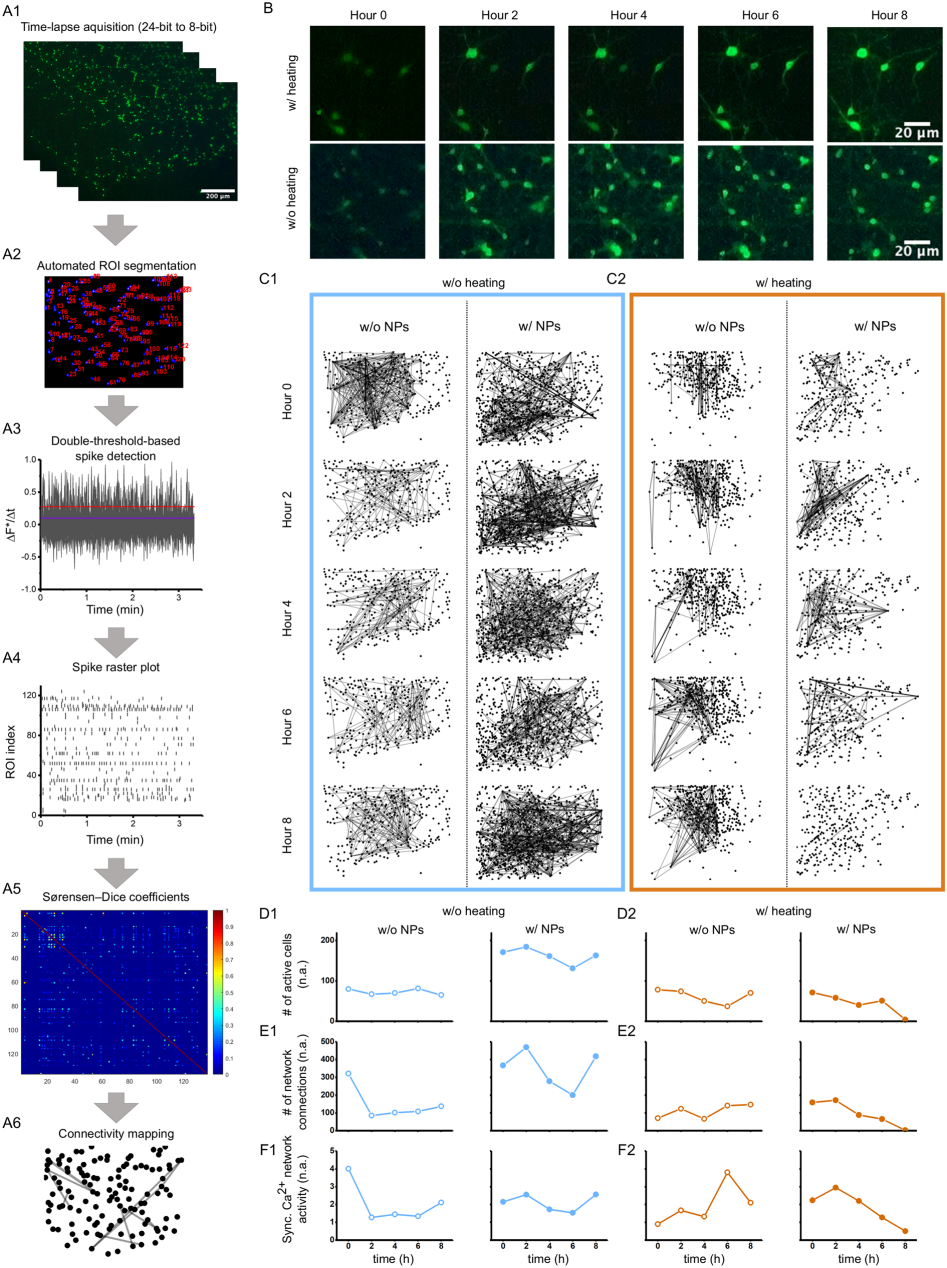
Chitosan-coated magnetic nanoparticles (NPs) impacts synchronicity in calcium signalling. (**A1**–**A6**) Image processing analysis to extract spatially resolved neuronal connectivity map based on primary cortical neurons labelled with Fluo-4. (**A1**) Time-lapse images show pseudo-coloured calcium activity captured with digital fluorescent microscopy. (**A2**) Automated image segmentation spatially selects individual regions of interest (ROIs) based on fluorescently active cell bodies. (**A3**) Changes in calcium activity get recorded over time and (**A4**) transferred into a calcium spike raster plot based on calcium influx. (**A5**) A cross-correlation matrix presents high (blue) and low (red) probability of temporally connected calcium spiking activity, which is used in (A6) to connect the ROIs in a connectivity map. (**B**) Representing fluorescent images of primary neurons incubated for 10 h in the digital imaging system. (**C1**–**C2**) Connectivity maps present changes in synchronous calcium spiking activity over an 8-h time span in cortical neurons grown for 9 days in vitro, independent of room temperature (w/o heating), physiological temperature (w/heating) and of incubation with chitosan-coated magnetic NPs (w/NPs). (**D1**–**F2**) Network analysis shows (**D1**–**D2**) number of active neurons under different temperature conditions, with and without NPs, (**E1**–**E2**) number of connections between ROIs under different temperature conditions, with and without NPs, and (**F1**–**F2**) active cell normalized count of connections under different temperature conditions, with and without NPs. These four plots indicate the activity of the network and how it changes under the different culture conditions.

Our fluorescent imaging system has shown the ability to capture long-term changes in calcium signalling based on a 1.7 mHz acquisition rate. The high-speed modalities of the digital microscope, however, can capture images up to 30 Hz, making it an ideal tool to study fast-scale communication patterns in cultured neuronal networks. To validate our system, we chose a commercial suspension of chitosan-coated fluorescent nanoparticles and monitored changes in fluorescent calcium signalling due to nanoparticle endocytosis. The uptake mechanism and behaviour of these nanoparticles with primary cortical neuron cultures have been extensively studied in previous work^28–30^, and therefore were chosen as a first validation experiment for our low-cost imaging system. To assess calcium-mediated communication patterns in neuronal networks, we grew dissociated cortical neurons up to 2 weeks and imaged their calcium activity based on Fluo-4 AM. Over an 8h time span, metabolic calcium activity was captured in intervals of 2h for 150 s with a 1 Hz acquisition rate. Representative images of Fluo-4 loaded neurons are shown in Fig. S8 and Fig. S9 (see supplementary file). Using a fully automated cell body segmentation in combination with double-threshold based calcium spike detection gave us spike raster plots for each sample set (Fig. 5A1–A4). From the spike raster plots, the cross-correlation between spike trains was computed using the Sørensen-Dice coefficient and visualized in a neuronal communication graph called connectivity map that shows synchronous calcium firing (Fig. 5A5–5A6)^31^.

Trending in neurobiology is the development of nanotools that can pass the blood–brain barrier, target drugs to specific brain cell types, or act as a local actuator for brain cell stimulation^32–34^. Previous studies have specifically explored the effectiveness of magnetic nanoparticle surface coatings on the uptake of the particles into neurons^30,33,35–37^. Understanding the temporal aspect of nanoparticle endocytosis, however, remains elusive as most uptake studies are endpoint measurements. To investigate temporal variations in nanoparticle endocytosis on the synchronicity of calcium firing, we exposed chitosan-coated magnetic nanoparticles to primary cortical neurons grown up to 9 days and imaged changes in calcium activity in our portable imaging system at 1 Hz for 150 s in 2 h time intervals over 8 h at RT and at 37 °C. Representative fluorescent images of Fluo-4 AM loaded neurons are shown for the two temperature conditions in Fig. 5B. Figure 5C1 and C2 show the influence of temperature and nanoparticle endocytosis on the connectivity maps. Without nanoparticle endocytosis, the connectivity maps indicate only minor changes in the location of vertices (active cell bodies), the number of active cell bodies show small cyclic activity remaining overall constant (Fig. 5D1), however, the number of edges reduces about 70% within the first 2 h (lines due to synchronic activity, Fig. 5E1). In contrast, when nanoparticles were administered, synchronic activity increased by about 43%, and a spatial shift of the active region occurred. Between 2 and 6 h, synchronic calcium activity w/NPs reduced by over 64%, before the number of network edges rises again about 120% (Fig. 5E1 and F1).

Comparing different temperatures, the total number of active cell bodies and edges are similar between physiological and room temperature (Fig. 5D1 versus 5E1, w/o NPs) as long as no nanoparticles were administered. The small oscillatory changes in calcium influx and efflux are mirrored in our previous temperature-sensation experiments, where the location of randomly selected cell bodies for a small number of cell groups (< 40 cells) does not impact calcium fluorometry (see supplementary data, Fig. S1 and Fig. S2). Imaging neurons under physiological temperature reveals a trending increase in synchronized calcium signalling with large amplitude oscillation, most-likely correlating with the spatial location of active vertices within the network (Fig. 5C2, D2, E2, F2, w/o NPs). Incubating chitosan-coated nanoparticles with neurons under 37 °C impacted the spatial location of active vertices and the number of edges with an overall decreasing trend between 2 and 8 h. While neurons growing under physiological temperatures showed less synchronous calcium spiking over time (> 2 h) when incubated with chitosan-coated nanoparticles, individual cell spiking activity may still be high or increased as shown in previous studies (see also spike raster plots at 6 h in supplementary data, Fig. S3 and Fig. S4)^29^. Furthermore, we noted that the incubation of chitosan-coated nanoparticles within the first 2 h increased synchronicity independent on the temperature.

The demonstrated capability of our system to assess communication patterns within living neural networks based on fast-scale low-cost calcium fluorometry goes beyond often used cell migration and cell viability assessments^7,8,38–40^. It advances small scale and portable imaging technology^11^ and provides an affordable tool to low-resource communities to learn and study more about brain cell communication. Furthermore, it revealed extensive details about how temperature and time interplay with calcium signalling during nanoparticle endocytosis.

## Conclusions

With an emerging need to capture time-sensitive aspects of fluorescently labelled proteins and signals in mammalian cells, we have designed a low-cost, portable, live-cell fluorescent imaging system from off-the-shelf-components. The described imaging solution is capable of studying calcium dynamics in human embryonic kidney cell lines and primary cultures of embryonic cerebral neurons and can control culture conditions and temperature. Using fluorescent probes and proteins^41,42^ in combination with nanoparticle interactions in cells, the low-cost imaging system is suitable to make calcium fluorometry and the study of changes in the cellular macro- (millimetre scale) and microenvironment (sub-micrometre scale) accessible for low-resource environments and provides advanced neuroscience research tools for the classroom^43^. The imaging setup uses digital fluorescent microscopy to capture changes in cytosolic calcium levels across time with up to 30 Hz. The imaging system is a low-tech version of similar live-cell imaging platforms^8–11,38,44–47^. It was purposely kept at low-cost (< 2,000 US$) and required no additional computer drawing skills or access to 3D printing. Although the spatial, optical, and radiometric resolution of our imaging system is lower than that of a high-quality optical microscope, the system can resolve fluorescent signals down to 1.352 pixels per micrometre. The utilized digital imaging modality provided more uniformity and lower noise sensitivity across the imaging sample, which increases the robustness for image signal processing. Furthermore, the imaging system has been demonstrated to provide stable temperature control for cell biology studies. While our imaging system cannot replace super-resolution microscopy to study subcellular transport and interaction of nanoparticles with other organelles, it brings the capability to monitor fast-scale temporal changes within large-scale cell networks. Specifically, we have shown the utility of our system to derive a neuronal graph called a connectivity map that shows synchronous calcium firing. Furthermore, we demonstrated the utility of graph network analysis to derive connectivity maps and applied them to nanoparticle uptake studies, which revealed extensive details about how temperature and time interplay with calcium signalling during the endocytic process.

Overall, we have demonstrated here a portable, low-cost imaging system that allows us to assess communication patterns within neural network going beyond often used cell migration and cell viability assessment and which can be used as an affordable alternative to cost-intense microscopy in low-resource communities to learn and study more about brain cell communication.

## Methods

### Portable, live-cell fluorescent imaging system

An imaging system was designed to maintain constant physiological temperature and humidity control for long-term live-cell monitoring, using off the shelf elements for high reproducibility at a low-cost. The imaging system was assembled based on a small-scale bench-top incubator with digital temperature control (e.g., MyTemp™ mini digital incubator), a digital fluorescent microscope with coloured illumination (LED-based, e.g., Dino-Lite AM4115T-GRFBY), a cell culture sample holder, and a LED-based white light illumination. The digital fluorescent microscope is a glass-lens based mini microscope and can be operated at two different excitation wavelengths of 480 nm and 575 nm to monitor green and red fluorescent probes through a USB- connected desk laptop. The optical sensor in the digital microscope is a CMOS camera with 1280 × 1024 pixels resolution (1.3 megapixels) that can capture up to 30 frames per second (fps). A full comparison of our digital live-cell fluorescent imaging system against other portable, low-cost digital and low-cost traditional optical imaging systems can be found in Table S1 (see supplementary file). Further methods describing the quantification of the digital fluorescent microscope imaging characteristics, and a 48 h long-term live-cell validation experiment based on Normal *Rattus norvegicus* Kidney (NRK) epithelial cells is also presented in the supplementary data file.

### Primary cortical neuron and human embryonic kidney cell culture

To demonstrate the effect of temperature control of the imaging system, we chose human embryonic kidney (HEK) as they are well known to exhibit endogenous calcium channels^12^ and show high sensitivity to non-physiological temperatures^15,18,48^. HEK cells were cultured in mouse embryonic fibroblast (MEF, passage 18) media. When grown to 80% confluency, cells were trypsinated and reseeded into pre-coated 35 mm Petri dishes for the temperature sensation experiment and grown for 2 days. To test the robustness of our imaging system with neurons, we monitored calcium signalling in neural cultures grown from dissociated rat cortical neurons. In neurons, calcium fluorometry is an important imaging methodology to study neuronal cell and network signalling^1,20,21,24,25,49–52^. Rat cortical hemispheres were dissected from whole embryonic rat brains (E18, BrainBits) and dissociated with 10% (v/v) papain (Carica papaya, Roche) in Hibernate®-E (BrainBits) at 35 °C for 15 min. Dissociated cortical neurons were seeded at a cell concentration of 1 million cells per ml into PEI pre-coated 35 mm Petri dishes at a cell density of 180 cells/mm^2^ and were incubated (95% air, 5% CO_2_, 37 °C) in serum-free Neurobasal with 2% (v/v) serum-free B-27® and 1% (v/v) PenStrep antibiotics, and grown until day 8 in vitro. For calcium fluorometry, Fluo-4 AM with probenecid acid was loaded to the cells (1:1) and incubated for 60 min in a standard incubator (37 °C, 5% CO_2_) following vendor protocol (ThermoFisher). Somatic calcium dynamics were monitored at 0.1 frames per min, 1 s exposure time for 10 h with cyclic on/off LED-light 480 nm excitation. The digital microscope was set to 160×–220× magnification. Cells were either monitored without heat at room temperature (w/o heat), or at physiological temperature (37 °C, w/heat) in the temperature-controlled live-cell imaging system over 10 h.

### Live-cell nanomaterial fluorometry

At 9 days in vitro (DIV), Fluo-4 AM loaded cortical neurons were exposed to chitosan-coated fluorescent magnetic nanoparticles (5 × 10^11^^11^ NP per ml, Chemicell, core: 100 nm, hydrodynamic radius: 190 nm, Fig. S5) and monitored for live-cell fluorescent imaging over 10 h. Extensive characterization of the chitosan-coated NPs can be found in Tay, Kunze et al*.*^29^. Somatic calcium dynamics were recorded with LED-light 480 nm excitation at 1 fps, 1 s exposure time for 5 min in the incubator system without heat at room temperature (w/o heat), or at physiological temperature (37 °C, w/heat). During a 2-h interval, neurons were left without excitation and imaged again with the same parameters. This process was repeated three times for a total imaging time of 8 h. For control, fluorescent neurons without magnetic nanoparticles were monitored under the same imaging parameters with and without physiological temperature settings.

### Fluorometric image processing

Grey-scale time-lapse images (8-bit) were analysed by selecting multiple single-cell regions of interests (ROIs). Fluorescence signal distribution was extracted, and relative fluorescence (F_rel_) was plotted based on Eq. 1, where F_max_ is the maximal detected fluorescent signal in all images, $\overline{F}$ is the averaged fluorescent intensity per ROI, and *F*_*Bkg*_ denotes the background fluorescent signal.

1 $$F_{rel} = \frac{\Delta F}{{F_{max} }}{ } = \frac{{\overline{F} - F_{Bkg} }}{{F_{max} }}$$

Time-varying changes of somatic fluorescence (*F*_*pixel*_) were recorded and averaged fluorescent intensity $\overline{F}$ across each cellular ROI was calculated using Eq. 2. Equation 2 shows $n$ as the total number of pixels and *F*_*pixel*_ as the intensity value of each indexed pixel per ROI.

2 $$\overline{F} = \frac{{\mathop \sum \nolimits_{i = 1}^{n} F_{pixel} }}{n}$$

Second, $\overline{F}$ was normalized by the average background (*F*_*Bkg*_) for each frame resulting in F^*^ as shown in Eq. 3:

3 $$F^{*} = \frac{{\overline{F}}}{{F_{Bkg} }}$$

Third, a relative fluorescence change (${{\Delta F^{*} } \mathord{\left/ {\vphantom {{\Delta F^{*} } {\Delta t}}} \right. \kern-\nulldelimiterspace} {\Delta t}}$), where Δ*t* is the framerate^-1^ was used for subsequent calcium signalling analysis. Calcium spike events were distinguished based on calcium influx and efflux. For both a double threshold analysis was applied based on a static ($\frac{{\Delta F^{*} }}{\Delta t} > \pm 0.05$) and a varying threshold ($\frac{{\Delta F^{*} }}{\Delta t} > \pm 5{\text{x}}$ standard deviation). A calcium spike event was then set as a calcium influx for a positive amplitude above the highest positive threshold, and as a calcium efflux event for a negative amplitude below the smallest negative threshold. From these calcium events, raster plots and connectivity maps were generated (see supplementary data for more information).

## Supplementary information

Supplementary information.

## Data Availability

A supplementary information file is available and provides additional information about methodology, data analysis, data calibration, and pre-processed calcium data, including ten supplementary figures (Figure S1–S10) and one supplementary table (Table S1).
