# Supplementary material for: Low-cost calcium fluorometry for long-term nanoparticle studies in living cells
Source: Sci Rep. 2020 Jul 28;10:12568. doi: 10.1038/s41598-020-69412-1 (PMC7387557; doi:10.1038/s41598-020-69412-1)
Supplement: Supplementary file 1 — Supplementary information. [file 41598_2020_69412_MOESM1_ESM.pdf]

Supplementary Information

Low-cost calcium fluorometry for long-term nanoparticle studies in living cells

Connor L. Beck[1] (B.Sc.), Clark J. Hickman[1,#] (B.A.) and Dr. Anja Kunze[1]* (Ph.D.)

*Corresponding author: anja.kunze@montana.edu
[1]Department of Electrical and Computer Engineering, Montana State University, Bozeman, Montana 59717, United States
[#]Present address of C.H.: Department of Physics, North Carolina State University, Raleigh, North Carolina, 27695, United States

Abstract
This document contains further details about experimental methodology, additional representative images, and data analysis associated with the article mentioned above.

1. Methodology

Portable, live-cell fluorescent imaging system. An imaging system was designed to maintain constant physiological temperature and humidity control for long-term live-cell monitoring, using off the shelf elements for high reproducibility at a low cost. The imaging system was assembled based on a small-scale benchtop incubator with digital temperature control (e.g., MyTemp™ mini digital incubator), a digital fluorescent microscope with coloured illumination (LED-based, e.g., Dino-Lite AM4115T-GRFBY), a cell culture sample holder, and a LED-based white light illumination. Wiring of the white light and digital camera were routed through an access port at the back of the incubator and sealed with Parafilm wax. The digital fluorescent microscope is a glass-lens based mini microscope. It can be operated at two different excitation wavelengths of 480 nm and 575 nm to monitor green and red fluorescent probes through a USB-connected desk laptop. The optical sensor in the digital microscope is a CMOS camera with 1280 × 1024 pixels resolution (1.3 megapixels) that can capture up to 30 frames per second (fps). The imaging system is a low-tech version of similar live-cell imaging platforms [1-9]. It was purposely kept at low-cost (< 2,000 US$) and required no additional computer drawing skills or access to 3D printing. The live-cell fluorescent imaging system was designed to make calcium nano fluorometry in neurons attractive for low-resource environments and to provide advanced neuroscience research tools for classrooms [10]. A full comparison of our digital live-cell fluorescent imaging system against other portable, low-cost digital and low-cost traditional optical imaging systems can be found in Table S1 (see supplementary file).

Quantification of the digital fluorescent microscope imaging characteristics. To verify the optical properties of the digital microscope at the resolution of single-cell bodies, we imaged Dragon Green fluorescent beads (15.65 µm) diluted in sterile water (1% (v/v)). Imaging characteristics of the digital microscope (220x) were quantified based on shape and brightness uniformity of bead-derived fluorescent signals. They were analyzed using the signal-to-noise ratio (SNR), mean-absolute-error (MAE), peak signal-to-noise (PSNR), and root-mean-standard error (RMSE) using an ImageJ plugin by Sage *et al.* [11].

For image comparison, green-fluorescence was captured in a 24-bit RGB format and converted to 8-bit grey-scale. Properties of the digital microscope images were compared with images taken from the beads with an optical fluorescent microscope (Amscope, 20x, and 40x optical magnification with a 6-megapixel camera) as reference. Fluorescent beads were excited with 475 nm light. Batches of ten images of the beads were recorded from both the digital and the optical imaging system for statistical analysis.

Temperature sensation in human embryonic kidney cell culture. To demonstrate the effect of temperature control of the imaging system, we chose human embryonic kidney (HEK) as they are well known to exhibit endogenous calcium channels [12] and to show high sensitivity to non-physiological temperatures [13-15]. HEK cells were cultured in mouse embryonic fibroblast (MEF, passage 18) media. When grown to 80% confluency, cells were trypsinated and reseeded into pre-coated 35 mm Petri dishes for the temperature sensation experiment and grown for two days. For calcium fluorometry in HEK cells, Fluo-4 AM with probenecid acid was loaded to the cells (1:1) and incubated for 60 min in a standard incubator (37 ºC, 5% CO2) following vendor protocol (ThermoFisher). Afterwards, HEK cells were gently washed with pre-warmed MEF media and then returned to the incubator for two hours. For the temperature sensation experiment, calcium fluorescent HEK cells were placed into the live-cell fluorescent imaging system, and somatic calcium dynamics were monitored at 0.1 frames per min, 1 s exposure time for 10 h. LED-light excitation at 480 nm was automatically controlled and switched on and off 10 s prior to image capture. The digital microscope was set to 160X magnification. HEK cells were either monitored without heat at room temperature (w/o heat), or at physiological temperature (37 °C, w/ heat) in the temperature-controlled live-cell imaging system.

Temperature sensation in primary cortical neuron cultures. Calcium fluorometry is an important imaging methodology to study neuronal cell and network signalling [16-24]. To test the robustness of our imaging system with neurons, we monitored calcium signalling in neural cultures grown from dissociated rat cortical neurons. Rat cortical hemispheres were dissected from whole embryonic rat brains (E18, BrainBits) and dissociated with 10% (v/v) papain (Carica papaya, Roche) in Hibernate®-E (BrainBits) at 35 °C for 15 min. The dissociated cortical neurons were centrifuged (6 min, 600 rpm, at room temperature) and seeded at a cell concentration of 1 million cells per ml into 35 mm Petri dishes. Petri dishes were coated with 0.05% (v/v) polyethyleneimine (PEI) for two hours and washed three times with phosphate-buffered solution (PBS) before cell seeding. Cortical neurons were seeded at a cell density of 180 cells/mm^2 and incubated (95% air, 5% CO2, 37 °C) in Neurobasal serum-free with 2% (v/v) serum-free B-27® and 1% (v/v) PenStrep antibiotics and grown until day 8 *in vitro*. For calcium fluorometry in the neuronal cultures, Fluo-4 AM with probenecid acid was loaded to the cells (1:1) and incubated for 60 min in a standard incubator (37 ºC, 5% CO2) following vendor protocol (ThermoFisher). For the temperature sensation experiment, calcium fluorescent neuron cultures were placed into the live-cell fluorescent imaging system, and somatic calcium dynamics were monitored at 0.1 frames per min, 1 s exposure time for 10 h with cyclic on/off LED-light 480 nm excitation, identical to the HEK cell experiment. Neuronal cells were either monitored without heat at room temperature (w/o heat), or at physiological temperature (37 °C, w/ heat) in the temperature-controlled live-cell imaging system over ten hours.

Multi-cell transient fluorometry. For both HEK and neuronal cells, 8-bit grey-scale time-lapse images were analyzed in sequence based on selecting multiple single-cell regions of interests. For each single-cell region of interest (ROI), fluorescence signal distribution was extracted, and relative fluorescence (F_{rel}) was

plotted based on equation 1, where F_{max} is the maximal detected fluorescent signal in all images, $\bar{F}$ is the averaged fluorescent intensity per ROI, and F_{Bkg} is denoted as the background fluorescent signal.

$$F_{rel} = \frac{\Delta F}{F_{max}} = \frac{\bar{F} - F_{Bkg}}{F_{max}} \quad eq.1$$

Live-cell nano fluorometry. At 9 days *in vitro* (DIV) cortical neurons were loaded with Fluo-4 AM for 60 min and gently washed as described above. Then chitosan-coated magnetic nanoparticles (5×10^{11} NP per ml, Chemicell, core: 100 nm, hydrodynamic radius: 190 nm, Fig. S5) were added to the calcium fluorescent living neurons and placed into the live-cell fluorescent imaging system for further live-cell fluorescent monitoring. Extensive characterization of the chitosan-coated NPs can be found in Tay, Kunze *et al.* [25]. Somatic calcium dynamics were recorded with LED-light 480nm excitation at 1 fps, 1 s exposure time for 5 minutes in the incubator system without heat at room temperature (w/o heat), or at physiological temperature (37 °C, w/ heat). During a two-hour interval, neurons were left without excitation and imaged again with the same parameters. This process was repeated three times for a total imaging time of 8 h. For control, fluorescent neurons without magnetic nanoparticles were monitored under the same imaging parameters with and without physiological temperature settings.

Calcium spike event detection and synchronous network activity mapping. Fluorescent images acquired in our live-cell imaging system were converted from their native '.wmv' video format to '.tiff' using FFmpeg. All image analysis was performed in MATLAB 2019A, and final graphical data were plotted using Origin Lab 2018b.

First, the tiff-based image stack was converted into an 8-bit image stack, and single-cell bodies were segmented and saved as individual regions of interests (ROIs) with their corresponding spatial x, y coordinates. Time-varying changes of somatic fluorescence (F_{pixel}) were recorded and saved as a time series data with averaged fluorescent intensity values across the pixels in each ROI. Equation 2 shows n as the total number of pixels within the ROI and F_{pixel} as the intensity value of each indexed pixel in the ROI and $\bar{F}$ as the averaged fluorescence intensity per ROI.

$$\bar{F} = \frac{\sum_{i=1}^{n} F_{pixel}}{n} \quad eq.2$$

Second, $\bar{F}$ was normalized by the average background (F_{Bkg}) for each frame resulting in F^* as shown in equation 3:

$$F^* = \frac{\bar{F}}{F_{Bkg}} \quad eq.3$$

Third, the rate of relative fluorescence change ($\Delta F^*/\Delta t$), where Δt is the framerate^{-1} was used for subsequent calcium signal analysis (spike event detection, signal correlation, and synchronous network activity mapping). Calcium spike events were distinguished based on calcium influx and efflux. For both a double threshold analysis was applied based on a static ($\frac{\Delta F^*}{\Delta t} > \pm 0.05$) and a varying threshold ($\frac{\Delta F^*}{\Delta t} >$ ±5x standard deviation). A calcium spike event was then set as a calcium influx event for a positive amplitude above the highest positive threshold, and as a calcium efflux event for a negative amplitude below the

smallest negative threshold. From these calcium events, raster plots were generated, showing either influx, efflux or both event types.

Pathological calcium events were analyzed separately from transient calcium dynamics. If a cell body exhibited a high cytosolic influx in calcium followed by a substantial efflux, this event might indicate a relation of calcium signalling with apoptosis or necrosis. These calcium events were identified by a systematic scan of the time-varying fluorescent data for a single influx followed by single efflux events. Next, the time delay (ΔT) between the influx and efflux event was extracted.

Calcium raster plots were compared for signal correlation and used to derive a connectivity map based on synchronous spiking activity between individual cell bodies. Only cell bodies exhibiting multiple positive or negative peaks were analyzed. Network nodes in our connectivity map were defined by the exact coordinates of segmented active cell bodies (at least one spike event occurred within the segmented ROI). Transient calcium spiking events were compared for a synchronic pair-wise occurrence and mapped onto a cross-correlation matrix in MATLAB 2019A. Calcium signal cross-correlation was determined based on computing the Sørensen-Dice similarity coefficient. For Dice-coefficients between 0.5 and 0.9, cells were assumed to be weakly connected, and a line with a transparency value of 40% and 5 pixels in width was drawn between their corresponding ROIs. For Dice-coefficients larger than 0.9, cells were assumed to be strongly connected, and a line with a transparency value of 40% and 7 pixels in width was drawn between their corresponding ROIs.

Cell network analysis. To compare macro and micro environmental effects on the neural network activity and performance, we further quantified three parameters for each individual network and reported their change over time. The three parameters are the number of active cells, the number of synchronous network connections, and the number of calcium spike events. Cells were counted as active if at least one calcium spike event had been identified from this indexed ROI throughout the recording period. The number of synchronous network connections was summed for each synchronously active ROI pair. Calcium event activity was computed based on the count of network connections normalized by the count of active cell bodies.

Long-term validation of cell viability.

To determine toxicity levels upon long-term imaging, we cultured Normal *Rattus norvegicus* Kidney (NRK, ATCC® CRL-6509™) epithelial cells in the imaging incubator for up to 48 h. Passage 6 NRK cells were detached from tissue culture flasks with 0.25% trypsin, 0.53 EDTA solution (2 min) and dispensed in new culture flasks at a subcultivation ratio of 1:6 (90% Dulbecco's Modified Eagle's Medium, 10% fetal bovine serum (v/v)). Culture flasks were placed in both the imaging incubator and a standard reference incubator for 48 hours. We performed bright field imaging at 0 h, 24 h, and 48 h. Live-dead staining was implemented by adding 4 µL of 3,3′-dioctadecyloxacarbocyanine (DiOC18) in 1 mL of culture medium at 24 h and 2 µL propidium iodide in 1 mL culture medium at 48 h followed by 5 min incubation at 37 °C. Cell viability (CV) was imaged using fluorescent microscopy and computed based on equation 4.

$$CV = \frac{\#\, green\ cells}{\#\, green\ cells + \#\, red\ cells} \cdot 100\ \% \quad eq. 4$$

2. Impact of single-cell sample selection in analyzing digital live-cell fluorescent images during long-term image acquisition

Heterogeneity in calcium signalling within cell cultures may impact average calcium intensity plots. Within our data set, the MATLAB algorithm randomly selects single cell bodies. Figure S1 A1 – A2 show the distribution of normalized calcium intensity over time for three different samples with a fixed number of randomly selected single-cell bodies (n_{cell} = 10). The plots only show minor differences in average intensity distribution. When accumulated over time, the data sets are not statistically different (Fig. S1 B).

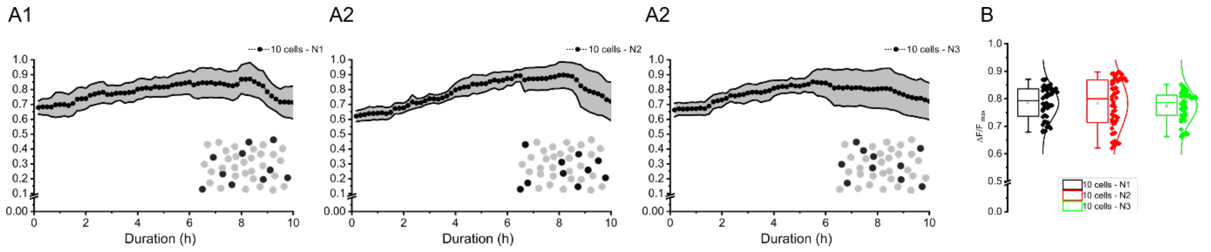

Figure S1: Averaged calcium intensity of ten randomly selected neuronal cell bodies from the same neuronal cell population. (A1-2) Calcium intensity distribution over 1 h in three different sample sets shows averaged calcium activity of ten randomly selected neuronal cell bodies. (B) Boxplots show the accumulated calcium intensity distribution of the three dependent sample sets.

Decreasing the fixed number of randomly selected cell bodies from 10 to 5, as well as increase the number from 10 to 20, resulting in a similar average intensity over time. However, for a fixed number of 40 cells, we observed a significant shift in average signal intensity (Fig. S2). This artefact may be due to differences in sub neuronal cell types, differences in metabolism, or calcium signalling heterogeneity in neuronal networks. To exclude this artefact in our temperature experiments, we compared smaller sample sizes. However, we also used this artefact as a motivation to evaluate calcium signalling synchronicity over time in response to nanoparticle uptake.

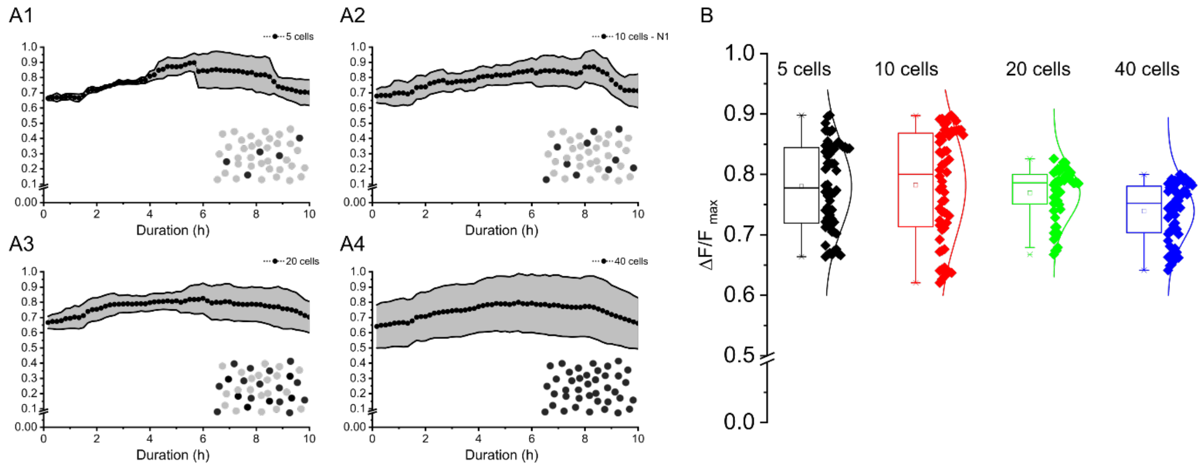

Figure S2: Sample-size effect in averaged calcium intensity plots. (A1-4) Averaged calcium intensity distribution over 10 hours in four different sample sets containing varying amounts of randomly selected single-cell bodies. Averaged plots for (A1) five, (A2) ten, (A3) twenty, and (A4) forty randomly selected cell bodies. (B) The boxplot shows the cumulated averaged calcium intensity distribution for the four different sample sets.

3. Calcium fluorometry derived from short-term digital live-cell fluorescent imaging.

Figure S3 and Figure S4 show comprehensive calcium data sets which include the rate of fluorescent change over time, extracted raster plots highlighting calcium influx and efflux events, and resulting cell-to-cell cross-correlation matrices from Fluo-4 loaded neurons which were recorded without temperature control (at room temperature, Fig. S3) and with temperature control at the physiological level (Fig. S4).

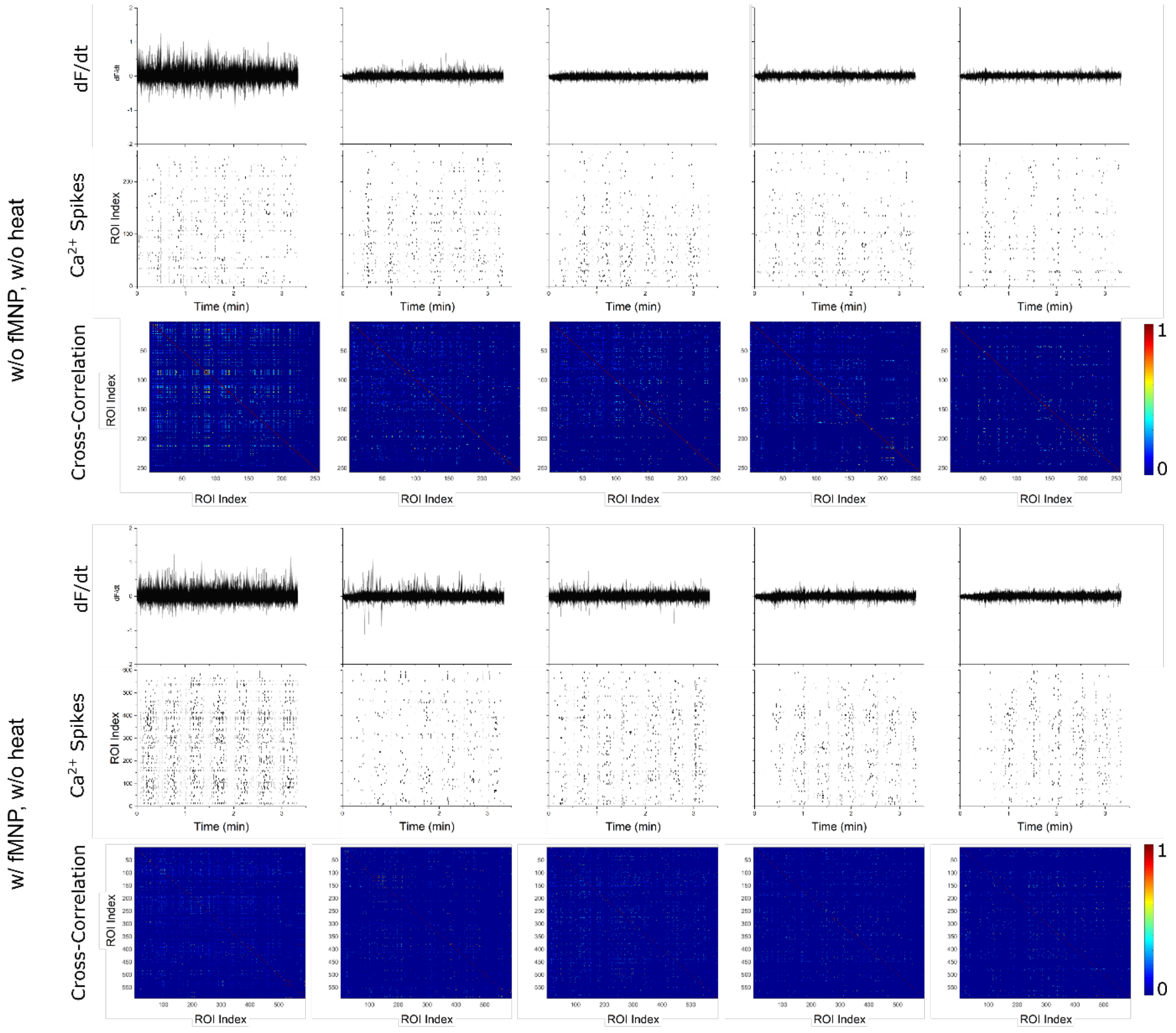

Figure S3: Calcium data sets were obtained in our study under room temperature. Each panel consists of a rate of change in calcium intensity recording cumulated over the entire recording time (first row), which was used to extract calcium signal spiking (influx and efflux events). Time-correlation between each calcium event resulted in a cross-correlation matrix, where blue colours indicate a low probability, and red colours indicate a high probability of correlated calcium events. Each column shows calcium signal recording at 0 h, 2 h, 4 h, 6 h, and 8 h. Calcium signals were recorded at 1 fps for 150 s.

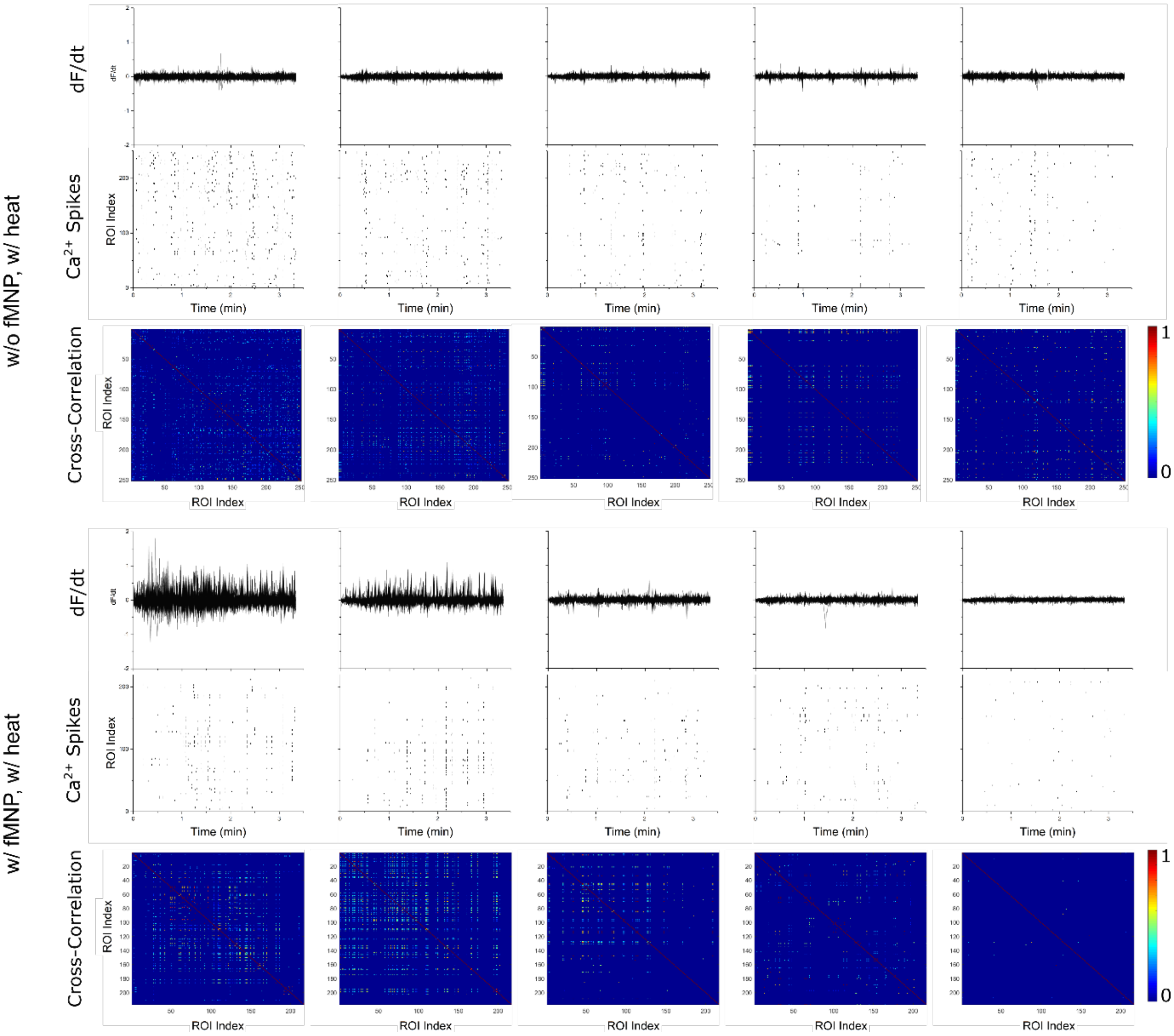

Figure S4: Calcium data sets were acquired in the digital live-cell fluorescent imaging system under heated (37 °C) incubation. Each panel consists of a rate of change in calcium intensity recording cumulated over the entire recording time (first row), which was used to extract calcium signal spiking events (influx and efflux). Time-correlation between each calcium event resulted in a cross-correlation matrix, where blue colours indicate a low probability, and red colours indicate a high probability of correlated calcium events. Each column shows calcium signal recording at 0 h, 2 h, 4 h, 6 h, and 8 h. Calcium signals were captured at 1 fps for 150 s.

5. Nanoparticle sizing with dynamic light scattering (DLS)

Incubating primary neuron cultures with nanomaterials has been shown to impact calcium signalling in previous studies [25-27]. To validate the capturing of similar calcium events in the digital live-cell imaging system, we incubated primary cortical neurons with chitosan-coated superparamagnetic nanoparticles. Figure S5 shows the size distribution of the hydrodynamic radius of the utilized nanoparticles measure in Neurobasal.

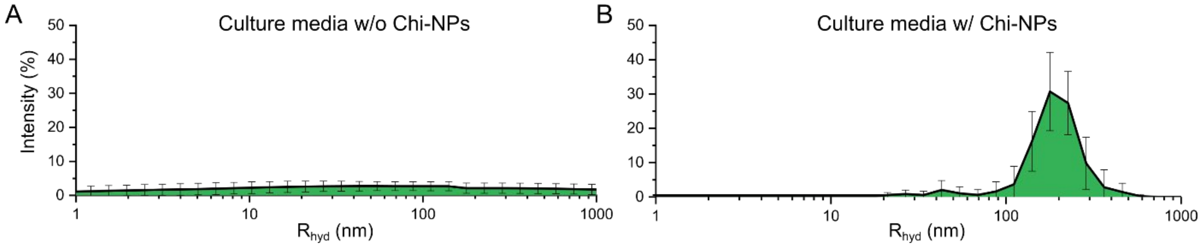

Figure S5: Nanoparticle dimension measured using dynamic light scattering (DLS). (A) Particle size and distribution in Neurobasal cell culture media. The uniform small-scale distribution indicates no particles in control (w/o Chi-NPs), small variations point towards the measurement error of the DLS instrument. No particles were detected. (B) Neurobasal culture medium spiked with chitosan-coated fluorescent superparamagnetic nanoparticles shows particles with a hydrodynamic radius (R_{hyd}) around 180 nm.

6. Supplementary characterization of key imaging parameters.

Histogram plots were used to assess image contrast for monitoring grey-scale, green, and red fluorescent images captured with the digital fluorescent microscope. Figure S6 shows the histogram distribution associated with the representative false-colour and grey-scale images.

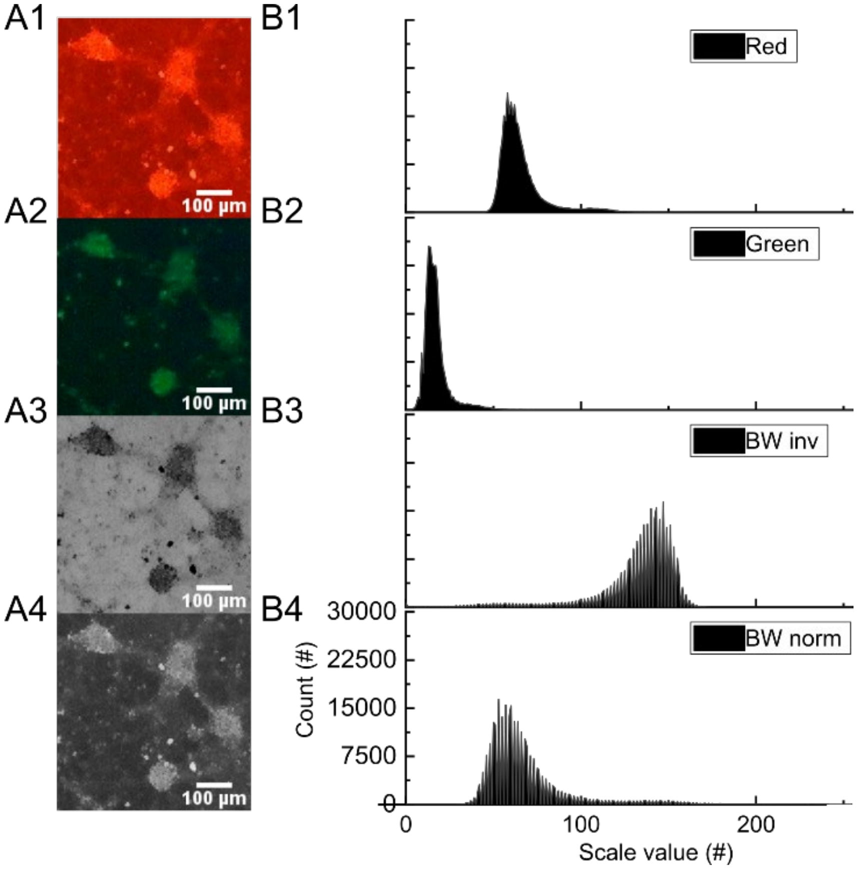

Figure S6: Histogram plots of 8-bit colour and grey-scale images were taken with the digital microscope. Images show clusters of primary cortical neurons grown for two weeks under standard conditions in a Petri dish. (A1) Neurons were loaded with a red-fluorescent mitotracker dye. 8-bit colour image. Yellow = high, dark-red = low fluorescent concentration. (A2) Neurons were loaded with the green-fluorescent calcium dye. 8-bit colour image. Green = high, black = low fluorescent concentration. (A3) Grey-scale brightfield image. Dark grey = high, light grey = low. (A4) Inverted Grey-scale brightfield image. Dark grey = low, light grey = high. (B1-B4) Associated histogram plots for the shown images. All images and data shown here are associated with Figure 1.

Although the image resolution of the digital fluorescent microscope is lower in comparison to images taken with a traditional optical fluorescent microscope, image contrast, as shown in the histogram plots based on green fluorescence in Figure S7, remains similar.

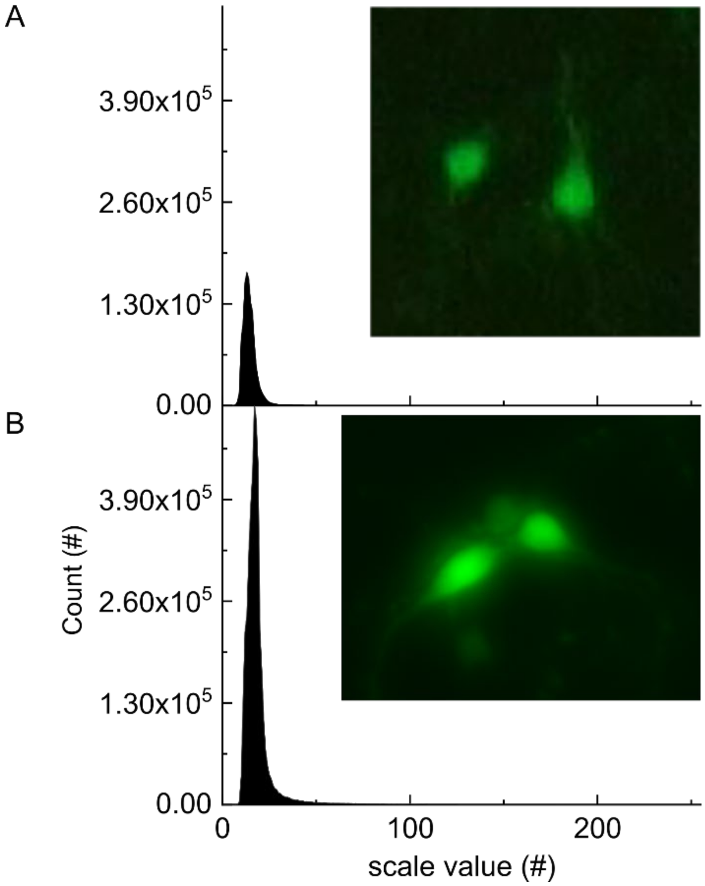

Figure S7: Histogram plots were extracted from 8-bit green-colour fluorescent images which were taken with (A) the portable digital fluorescent imaging system, and with (B) the non-portable traditional optical fluorescent microscope (20x objective). Images show single primary cortical neurons that were loaded with Fluo-4 AM and grown for two weeks under standard culture conditions in a Petri dish.

7. Representative fluorescent images captured during the temperature sensation experiment.

Figure S8 shows representative fluorescent images extracted from the video recordings done with the digital live-cell fluorescent imaging system to validate long-term image acquisition and portability. HEK cells were monitored in a lab-extern cell culture facility. Primary neuron cultures were monitored in our lab. Figure S9 shows representative fluorescent images extracted from the video recordings done with the digital live-cell fluorescent imaging system to validate short-term image acquisition using primary cortical neurons.

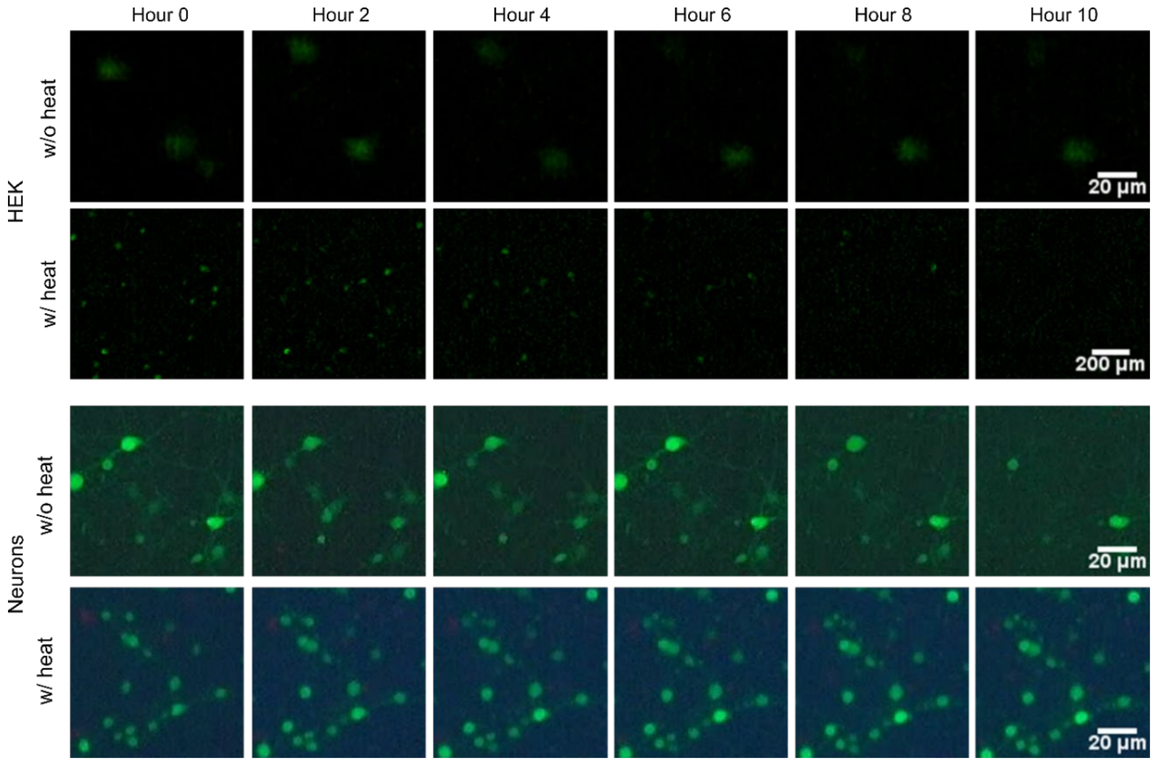

Figure S8: Representative calcium images of Fluo-4 loaded human embryonic kidney (HEK) cells and primary cortical neurons. Green-fluorescent signals were monitored over time in the digital imaging system without (w/o heat) and with heat (w/ heat) setting to physiological temperature (37 °C).

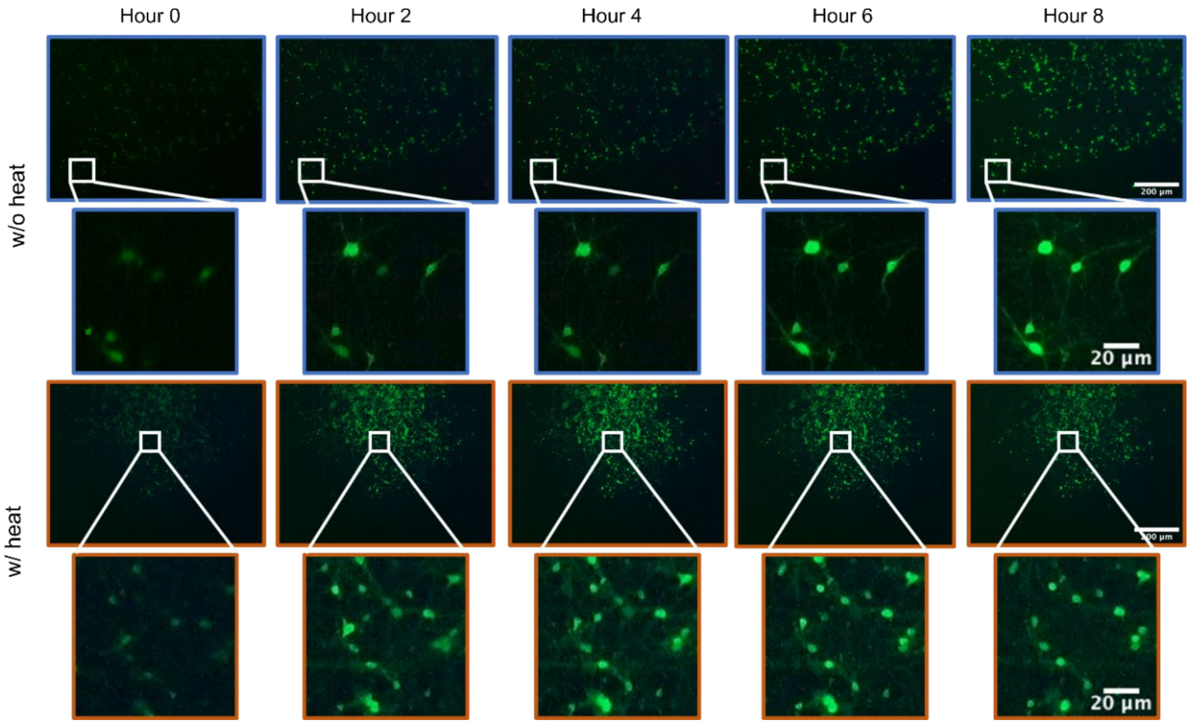

Figure S9: Representative calcium images of Fluo-4 primary cortical neurons. Green-fluorescent signals were monitored over time in the digital imaging system without (w/o heat) and with heat (w/ heat) setting to physiological temperature (37 °C).

8. Long-term live-cell validation after 48 h incubation

Figure S10A (right panel) shows NRK cells growing in culture flasks in the portable imaging incubator system at the start (0 h) and after 24 h and 48 h. Comparing NRK cells grown in the portable incubator with a standard incubator system yields no significant differences in their cell density, proliferation rate, morphology and cell viability (Fig. S10A-B). After 48 h, fluorescent imaging displayed cell viability of 98.7% for the established incubation system and 98.6% for the portable incubation system based on Live/Dead staining (Fig. S10C, Live: green = $DiOC_{18}$, Dead: red = Propidium Iodide). Hence, the portable imaging incubator can support live-cell imaging in a remote setting over up two days, if needed.

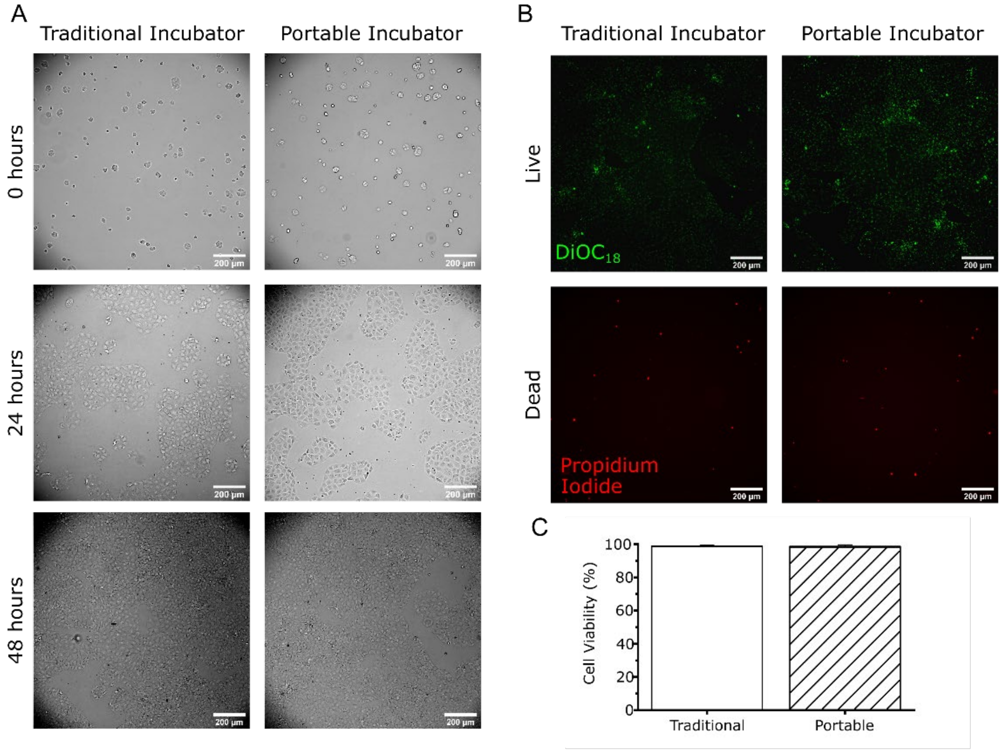

Figure S10: Comparing NRK live-cell cultures between standard and portable incubator systems. (A) Brightfield images were taken of NRK cells grown up to 48 h under physiological temperature (37 °C, w/ heat). (B) Fluorescent images show Live/Dead stained NRK cells after 48 h. Note: NRK cells were stained with the Live/Dead dyes 24 h before imaging. (C) Extracted live cell viability index based on the number of living cells over the number of totally stained cells confirms the long-term cell viability in the portable incubation system.

9. Comparison of low-cost imaging systems

Table S1 compares quantitative measures of the assembled and used low-cost digital live-cell imaging systems against other reported low-cost digital and traditional optical imaging systems. All digital imaging systems have a clear cost advantage with varying long-term imaging capabilities; however, they lack behind in spatial resolution in comparison to traditional optical systems.

Table S1: Comparison of low-cost microscope imaging systems

	Digital system, used	Walzik [8]	Rajan [5]	Hasan [7]	Zhang [28]	Gurkan [1]	Jin [9]	Low-cost traditional optical systems
**Cost (US$)**	1,453	~ 1,400	n.r.	358	10	184	~ 300	~ 15,000
**Incubator**	Yes	Yes	Yes	Yes	No	Yes	Yes	No
**Fluorescent acquisition**	Yes	No	No	No	Yes	No	Yes	Yes
**Size (dm^3/kg)**	70/ 8.1	10.6/ 3.6	n.r./ n.r	n.r./ 0.13	0.13/ 6.5e-2	23.1/ n.r.	1.4/ n.r.	~ 80/ > 20
**Real-time computer interface**	Yes	Yes	Yes	Yes	Yes	Yes	Yes	Yes
**Frame rate (fps)**	30	--	40-60	--	30	30	--	25
**Resolution (pixel/µm)**	1.352	0.571	0.7	--	0.5	0.56	0.33	1.25 (10x) 3 (20x)
**FOV (µm @ highest resolution)**	950 x 750	385 x 385	162 x 162	n.r.	130 x 105	685 x 385	15330 x 12270	668 x 668 (20x)
**Long-term imaging capabilities**	48 h	48 h	> 12 h	n.r.	n.r.	> 48 h	12 h	< 2 h

FOV = Field-of-view, n.r. = not reported

References

1 Gürkan, G. & Gürkan, K. Incu-Stream 1.0: An Open-Hardware Live-Cell Imaging System Based on Inverted Brightfield Microscopy and Automated Mechanical Scanning for Real-Time and Long-Term Imaging of Microplates in Incubator. *IEEE Access* **7**, 58764-58779, doi:10.1109/ACCESS.2019.2914958 (2019).

2 Linsley, J. W. *et al.* Automated four-dimensional long term imaging enables single cell tracking within organotypic brain slices to study neurodevelopment and degeneration. *Communications Biology* **2**, 155, doi:10.1038/s42003-019-0411-9 (2019).

3 Ragazzini, G., Mescola, A., Corsi, L. & Alessandrini, A. Fabrication of a low-cost on-stage cell incubator with full automation. *Journal of Biological Education* **53**, 165-173, doi:10.1080/00219266.2018.1451772 (2019).

4 Yang, K. *et al.* Recent development of portable imaging platforms for cell-based assays. *Biosensors and Bioelectronics* **124-125**, 150-160, doi:https://doi.org/10.1016/j.bios.2018.10.024 (2019).

5 Rajan, D. K. *et al.* A Portable Live-Cell Imaging System With an Invert-Upright-Convertible Architecture and a Mini-Bioreactor for Long-Term Simultaneous Cell Imaging, Chemical Sensing, and Electrophysiological Recording. *IEEE Access* **6**, 11063-11075, doi:10.1109/ACCESS.2018.2804378 (2018).

6 Schneidereit, D., Kraus, L., Meier, J. C., Friedrich, O. & Gilbert, D. F. Step-by-step guide to building an inexpensive 3D printed motorized positioning stage for automated high-content screening microscopy. *Biosensors and Bioelectronics* **92**, 472-481, doi:https://doi.org/10.1016/j.bios.2016.10.078 (2017).

7 Hasan, M. M., Alam, M. W., Wahid, K. A., Miah, S. & Lukong, K. E. A Low-Cost Digital Microscope with Real-Time Fluorescent Imaging Capability. *PLOS ONE* **11**, e0167863, doi:10.1371/journal.pone.0167863 (2016).

8 Walzik, M. P. *et al.* A portable low-cost long-term live-cell imaging platform for biomedical research and education. *Biosensors and Bioelectronics* **64**, 639-649, doi:https://doi.org/10.1016/j.bios.2014.09.061 (2015).

9 Jin, D. *et al.* Compact Wireless Microscope for In-Situ Time Course Study of Large Scale Cell Dynamics within an Incubator. *Scientific Reports* **5**, 18483, doi:10.1038/srep18483 (2015).

10 Gage, G. J. The Case for Neuroscience Research in the Classroom. *Neuron* **102**, 914-917, doi:10.1016/j.neuron.2019.04.007 (2019).

11 Sage, D. & Unser, M. Teaching image-processing programming in Java. *IEEE Signal Processing Magazine* **20**, 43-52, doi:10.1109/MSP.2003.1253553 (2003).

12 Berjukow, S. *et al.* Endogenous calcium channels in human embryonic kidney (HEK293) cells. *British journal of pharmacology* **118**, 748-754 (1996).

13 Iftinca, M. *et al.* Temperature dependence of T-type calcium channel gating. *Neuroscience* **142**, 1031-1042, doi:https://doi.org/10.1016/j.neuroscience.2006.07.010 (2006).

14 Peier, A. M. *et al.* A TRP Channel that Senses Cold Stimuli and Menthol. *Cell* **108**, 705-715, doi:https://doi.org/10.1016/S0092-8674(02)00652-9 (2002).

15 Xu, H. *et al.* TRPV3 is a calcium-permeable temperature-sensitive cation channel. *Nature* **418**, 181, doi:10.1038/nature00882 https://www.nature.com/articles/nature00882#supplementary-information (2002).

16 Jayakumar, S. & Hasan, G. Neuronal Calcium Signalling in Metabolic Regulation and Adaptation to Nutrient Stress. *Frontiers in Neural Circuits* **12**, 25 (2018).

17 Bahar, E., Kim, H. & Yoon, H. ER Stress-Mediated Signalling: Action Potential and Ca(2+) as Key Players. *Int J Mol Sci* **17**, 1558, doi:10.3390/ijms17091558 (2016).

18 Patel, T. P., Man, K., Firestein, B. L. & Meaney, D. F. Automated quantification of neuronal networks and single-cell calcium dynamics using calcium imaging. *Journal of neuroscience methods* **243**, 26-38, doi:10.1016/j.jneumeth.2015.01.020 (2015).

19 Tibau, E., Valencia, M. & Soriano, J. Identification of neuronal network properties from the spectral analysis of calcium imaging signals in neuronal cultures. *Frontiers in Neural Circuits* **7**, 199 (2013).
20 Grienberger, C. & Konnerth, A. Imaging Calcium in Neurons. *Neuron* **73**, 862-885, doi:https://doi.org/10.1016/j.neuron.2012.02.011 (2012).
21 Gleichmann, M. & Mattson, M. P. Neuronal calcium homeostasis and dysregulation. *Antioxid Redox Signal* **14**, 1261-1273, doi:10.1089/ars.2010.3386 (2011).
22 Zündorf, G. & Reiser, G. Calcium dysregulation and homeostasis of neural calcium in the molecular mechanisms of neurodegenerative diseases provide multiple targets for neuroprotection. *Antioxid Redox Signal* **14**, 1275-1288, doi:10.1089/ars.2010.3359 (2011).
23 Augustine, G. J., Santamaria, F. & Tanaka, K. Local Calcium Signalling in Neurons. *Neuron* **40**, 331-346, doi:https://doi.org/10.1016/S0896-6273(03)00639-1 (2003).
24 Tank, D. W., Sugimori, M., Connor, J. A. & Llinas, R. R. Spatially resolved calcium dynamics of mammalian Purkinje cells in cerebellar slice. *Science* **242**, 773 (1988).
25 Tay, A., Kunze, A., Murray, C. & Di Carlo, D. Induction of Calcium Influx in Cortical Neural Networks by Nanomagnetic Forces. *ACS Nano* **10**, 2331-2341, doi:10.1021/acsnano.5b07118 (2016).
26 Alivisatos, A. P. *et al.* Nanotools for Neuroscience and Brain Activity Mapping. *ACS Nano* **7**, 1850-1866, doi:10.1021/nn4012847 (2013).
27 Gahl, T. J. & Kunze, A. Force-Mediating Magnetic Nanoparticles to Engineer Neuronal Cell Function. *Frontiers in Neuroscience* **12**, 299 (2018).
28 Zhang, Y. S. *et al.* A cost-effective fluorescence mini-microscope for biomedical applications. *Lab on a Chip* **15**, 3661-3669, doi:10.1039/C5LC00666J (2015).
